# Supplementary material for: Emotion Evaluation and Response Slowing in a Non-Human Primate: New Directions for Cognitive Bias Measures of Animal Emotion?
Source: Behav Sci (Basel). 2016 Jan 11;6(1):2. doi: 10.3390/bs6010002 (PMC4810036; doi:10.3390/bs6010002)
Supplement: Supplementary File 1 [file behavsci-06-00002-s001.pdf]

Supplementary Materials: Emotion Evaluation and Response Slowing in a Non-Human Primate: New Directions for Cognitive Bias Measures of Animal Emotion?

Emily J. Bethell [1,*], Amanda Holmes [2,†], Ann MacLarnon [3,†] and Stuart Semple [3,†]

1. Supplementary Material for Methods

1.1. Veterinary Examination

The health check consisted of restraint for sedation by injection with Ketamine Hydrochloride (KHCl), followed by a routine veterinary inspection. Immediately prior to the veterinary health-check, the dedicated Veterinary Technologist (VT) entered the monkeys' enclosure with a trolley containing all equipment. The VT restrained the first monkey in his home cage using the squeeze-back mechanism and administered an intramuscular injection of Ketamine Hydrochloride (KHCl: 0.1 cc/kg). Once sedated, the monkey was removed from the cage for the veterinary examination (approximately 10 min duration per monkey). On return to the home cage each monkey was observed until he had recovered from the anaesthesia (approximately 15 min until mobile), and was then fed the daily food ration. The veterinary health-check consisted of weighing, inspection of nails, teeth and pelage and administration of a test for Tuberculosis bacteria. No further drugs were administered as routine. All monkeys on other medications were excluded from the study.

1.2. Baseline Phase

During the baseline phase monkeys were provided with additional enrichments in the homecage and extra care was taken to ensure that monkeys were not exposed to potential stressors (e.g., seeing the veterinarian).

Table S1. The distribution of trials which were classed as invalid and removed from the analysis (*i.e.*, trials which occurred sooner than 440ms or on which no response was made).

**Grp**	**ID**	**Responses <400 ms**						**Non-responses**						**Total**
		**Baseline**			**Stress**			**Baseline**			**Stress**			
		**Control**	**Direct Gaze**	**Averted Gaze**	**Control**	**Direct Gaze**	**Averted Gaze**	**Control**	**Direct Gaze**	**Averted Gaze**	**Control**	**Direct Gaze**	**Averted Gaze**	
	29C	-	-	1	-	6	-	-	-	-	-	-	-	7
1	C55	-	1	1	-	3	1	-	1	1	-	-	-	8
	16P	1	1	3	1	3	6	-	-	-	-	-	-	15
	AI73	2	2	-	1	-	1	3	11	9	1	7	3	40
	92R	8	14	15	9	20	19	-	-	-	-	-	-	85
2	27S	2	3	1	4	7	3	1	-	-	1	1	-	23
	66S	3	6	3	-	11	11	3	1	-	-	-	1	39
Total			67			106			30			14		217

1.3. Touchscreen Training

The monkeys were first trained to use the touch screen monitor. On the first day of training, each monkey was presented with a dummy version of the monitor covered in honey and leaves. Each time the monkey touched the screen to retrieve a leaf the experimenter (EB) manually sounded the reward tone that would be used in later sessions by pressing a key on a laptop. Training sessions lasted 20 min or until the monkey made 50 presses, whichever occurred sooner. Leaves and honey were reduced over successive days for each monkey according to his speed of learning, and the experimenter began to reward each correct touchscreen press by handing the monkey a food pellet. Once a monkey was touching the screen for food pellets training was transferred to the touchscreen monitor. A white square stimulus (40 mm × 40 mm) was shown at different points on the screen and screen touches that occurred within the frame of the white square were rewarded with automatic delivery of a pellet from the pellet dispenser. Trial duration was 60 s or until the monkey touched the stimulus at which point a rewarding tone sounded and a pellet was delivered automatically from the dispenser. If the monkey did not touch the stimulus then the next trial started after 60 s, with >8080 msec inter trial interval. Learning criterion was 30 correct presses in a 20 min window. The experimenter sat in with each monkey until he had completed three days at criterion with the touchscreen and appeared to be safe to work without close supervision (*i.e.*, he did not slap or hit the apparatus or show visible signs of frustration or aggression). The experimenter then observed all future sessions via a video link to the next room. Training continued, and the number of trials was increased for each monkey until he was performing 80 trials within a 40 min session. Concurrently, reward rate was reduced from 100% fixed ratio to 40% variable reward ratio to encourage animals to continue working as trial number increased. The tone was sounded for all correct touches. Criterion for entry to the study was 80 trials completed within 40 min on each of three successive days.

2. Supplementary material for Results

Table S2. The number of daily training sessions for monkeys to press the grey square presented at three locations on the touch screen.

ID	Age (yrs)	N Daily Sessions to Criterion
C55	24.70	16
29C	12.05	3
06H	9.9	13
16P	5.15	9
79T	3.65	4
92R	4.75	13
27S	4.66	12
66S	3.80	5
79S	3.70	11
AI73	3.60	4

Table S3. Performance during testing. Monkey identity, age at the start of the study, number of experimental trials completed during testing, proportion of the reward pellets eaten and whether the daily food ration was eaten at the end of the session (as measures of food motivation), and a summary of whether criteria for entry into the analysis were met.

			N Experimental Sessions Completed (≥80%)		Proportion of Pellets Eaten			Daily Food Ration Eaten			
	**ID**	**Age (yrs)**	**Baseline**	**Stress**	**Training**	**Baseline**	**Stress**	**Training**	**Baseline**	**Stress**	**Criteria Met? (yes/no)**
Grp 1	C55	24.70	2	2	0.99	0.95	0.99	✓	✓	✓	✓
	29C	12.05	2	2	0.98	0.99	1.00	✓	✓	✓	✓
	16P	5.15	2	2	1.00	1.00	1.00	✓	✓	✓	✓
	79T	3.65	0 *	-	0.92	-	-	✓	✓	✓	×
	AI73	3.60	1	1	0.85	0.91	0.89	✓	✓	✓	✓
Mean		9.83 ± 9.01	1.40 ± 0.89	1.75 ± 0.50	0.95 ± 0.06	0.96 ± 0.04	0.97 ± 0.05				n = 4
Grp 2	06H	9.90	0 *	2	1.00	-	1.00	✓	✓	✓	×
	92R	4.75	2	2	1.00	1.00	1.00	✓	✓	✓	✓
	27S	4.66	2	2	1.00	0.82	1.00	✓	✓	✓	✓
	66S	3.80	2	2	1.00	0.65	1.00	✓	✓	✓	✓
	79S	3.70	2	0 *	1.00	1.00	-	✓	✓	✓	×
Mean	*N* = 6	5.36 ± 2.58	1.60 ± 0.89	1.60 ± 0.89	1.00 ± 0.00	0.87 ± 0.17	1.00 ± 0.00				n = 3
Total	*N* = 12	7.39 ± 1.75	1.50 ± 0.23	1.40 ± 0.27	0.94 ± 0.04	0.89 ± 0.05	0.96 ± 0.03				n = 7

2.1. Non-Significant Results not Reported in the Text

2.1.1. Frequency of Responses on Control Trials

A 2 × 3 RMAnova was performed to determine whether stress-related arousal improves task performance (frequency of responses) when non-emotional control stimuli are shown. Data were proportion of responses on control trials, with within-subjects factors of testing condition (baseline versus stress) and screen location (left, central, right: Table S4). There were no main effects of either testing condition ($F_{1,6} = 2.273$, $p = 0.182$), nor screen location ($F_{2,12} = 0.000$, $p = 1.00$), nor interaction of location x testing condition ($F_{2,12} = 2.909$, $p = 0.093$).

Table S4. Proportion of responses made on control trials presented to the left, central and right visual fields.

	Baseline			Stress		
ID	Left	Central	Right	Left	Central	Right
C55	1.0	1.0	1.0	1.0	1.0	1.0
29C	1.0	1.0	1.0	1.0	1.0	1.0
16P	1.0	1.0	1.0	1.0	1.0	1.0
AI73	0.9	0.9	0.9	1.0	0.9	1.0
92R	1.0	1.0	1.0	1.0	1.0	1.0
27S	0.9	1.0	1.0	1.0	0.9	1.0
66S	0.9	1.0	0.8	1.0	1.0	1.0
Group $\overline{X}$	0.96 (±0.02)	0.99 (±0.01)	0.96 (±0.03)	1.00 (±0.00)	0.98 (±0.01)	1.00 (±0.00)

2.1.2. Latency to Respond on Control Trials

A 2 × 3 RmAnova was conducted to test the effect of stress-related arousal on control trials. Data were Log10RTs on control trials, with within-subjects factors of testing condition (baseline versus stress) and screen location (left, central and right). There was a main effect of testing condition ($F_{1,6} = 17.611$, $p = 0.006$: Figure S1). Monkeys were faster to respond on control trials in the stress condition than at baseline. There was no main effect of screen location ($F_{2,12} = 0.307$, $p = 0.741$), and no interaction of testing condition × screen location ($F_{2,12} = 1.561$, $p = 0.250$). This suggests there were no side biases in speed to respond. To give an indication of the range of response latencies, median untransformed RTs are shown in Table S5.

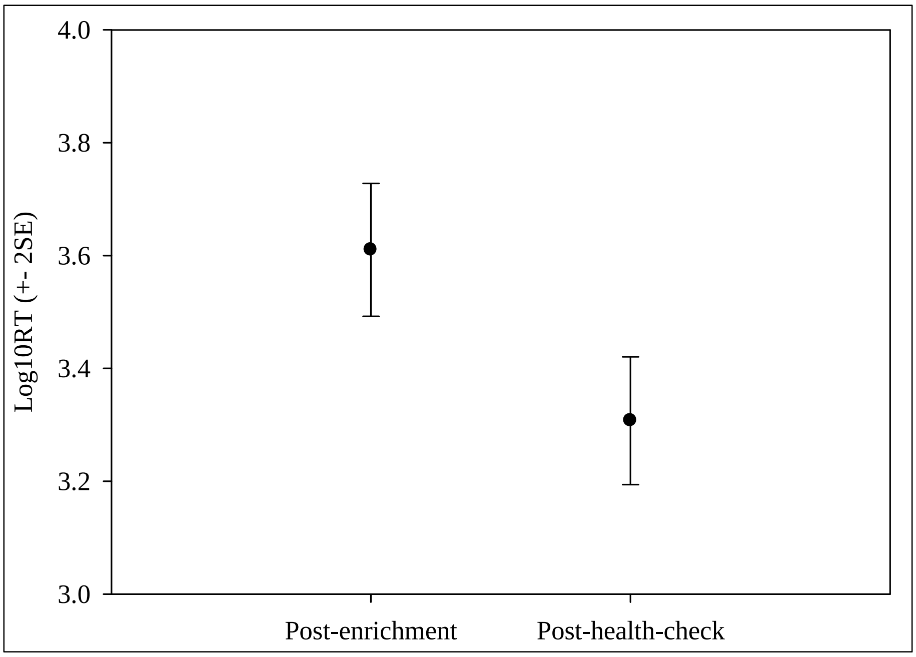

Figure S1. Log10RT for control trials in the baseline and stress conditions.

Table S5. Median latencies (ms) to respond to stimuli.

	Stress			Baseline		
**ID**	**Control**	**Direct Gaze**	**Averted Gaze**	**Control**	**Direct Gaze**	**Averted Gaze**
29C	2803	2470	3031	8327	4000	6202
C55	1409	5800	3513	4121	3876	3518
16P	874	1313	1399	1817	1910	2195
AI73	6973	10113	3147	16953	4927	13080
92R	2513	1180	2153	2447	1500	2160
27S	2607	1887	2120	4073	2593	5880
66S	1144	1237	1303	5901	1946	3543
$\overline{X}$	2618	3429	2381	6234	2964	5225
